# SNGH16 regulates cell autophagy to promote Sorafenib Resistance through suppressing miR‐23b‐3p via sponging EGR1 in hepatocellular carcinoma

**DOI:** 10.1002/cam4.3020

**Published:** 2020-04-23

**Authors:** Zhao Jing, Xiaoping Ye, Xiaojie Ma, Xiangrong Hu, Wenjun Yang, Junping Shi, Gongying Chen, Ling Gong

**Affiliations:** ^1^ Department of Radiation Oncology Hangzhou Cancer Hospital Hangzhou China; ^2^ Department of Liver Diseases The Affiliated Hospital of Hangzhou Normal University Hangzhou China; ^3^ Department of Pathology The Affiliated Hospital of Hangzhou Normal University Hangzhou China

**Keywords:** EGR1, hepatocellular carcinoma, MiR‐23b‐3p, SNHG16, sorafenib resistance

## Abstract

**Objective:**

Tumor cells could acquire drug resistance through cell autophagy. This study aimed to explore the role of SNHG16 in sorafenib‐resistant HCC cells and its mechanism with miR‐23b‐3p.

**Methods:**

The sorafenib‐resistant Hep3B cell model was established. The SNHG16 and miR‐23b‐3p gene expressions were determined in normal HCC and sorafenib‐resistant HCC tissues. Detection of the expression of SNHG16 and miR‐23b‐3p and its respective correlation with survival rate were performed. Target genes to SNHG16 and miR‐23b‐3p were predicted, and verified by dual‐fluorescent reporter assay. The effects of SNHG16 and miR‐23b‐3p on SNHG16, miR‐23b‐3p, EGR1 expression, viability, apoptosis as well as LC3II/LC3 expression in Hep3B and Hep3B/So cells were detected by qRT‐PCR, CCK‐8, flow cytometry, and western blot. In in vivo studies, the NOD/SCID mice model was established to explore the effects of Hep3B and Hep3B/So cells with inhibited SNHG16 or miR‐23b‐3p on tumor size, EGR1 expression, and autophagy.

**Results:**

High SNHG16 expression in HCC‐resistant tissues and low miR‐23b‐3p expression in all HCC tissues were detected, and the two were negatively correlated. Low SNHG16 and high miR‐23b‐3p were related to a high survival rate of HCC patients. Moreover, SNHG16 overexpression promoted Hep3B/So cell viability and autophagy, suppressed apoptosis by inhibiting miR‐23b‐3p expression through up‐regulating EGR1, however, the effect of si‐SNHG16 was opposite. In in vivo studies, miR‐23b‐3p inhibitor suppressed the high sorafenib sensitivity in Hep3B/So cells caused by SNHG16 silencing through promoting viability, autophagy, and suppressing apoptosis.

**Conclusion:**

SNHG16 promotes Hep3B/So cell viability, autophagy, and inhibits apoptosis to maintain its resistance to sorafenib through regulating the expression of miR‐23b‐3p via sponging EGR1.

## Graphical abstract

This study demonstrated the promoter role of SNHG16 played in regulating autophagy in Hep3B cells with sorafenib resistance, which further contribute to a stronger sorafenib resistance in Hep3B cells. Moreover, the above process implemented through regulating the expression of miR‐23b‐3p via sponging EGR1.

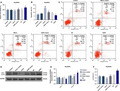

## 1 INTRODUCTION

Hepatocellular carcinoma (HCC) is one of the most frequent cancers in China. HCC has a high mortality among patient, as patients with the disease are often diagnosed in the advanced stage of HCC, on which current therapies are less effective in treating HCC. It is believed that virus infection of hepatitis B and hepatitis C,^1^, ^2^ which was realized through the regulation of cell proliferation and apoptosis, is the mechanism underlying the tumorigenesis of HCC. Currently, the main chemical therapies of HCC are oxaliplatin, doxorubicin, fluorouracil, etc.^3^, ^4^ However, such conventional treatment to patients with advanced HCC has its limitations, and is often accompanied with severe side effects. Soafinib is a new type of multitargeted drug, and it is mainly used for tumor treatment. As reported by Saidak, sorafenib suppressed progression of HCC via suppressing the Raf/MEK/ERK and PI3K/Akt/MTOR signaling pathways to inhibit the proliferation of tumor cells.^5^ Moreover, sorafenib was also reported to have the ability to inhibit VEGFR and PDGF receptor to further block the formation of tumor blood vessels, therefore indirectly suppressing tumor growth.^6^, ^7^ In advanced HCC, sorafenib could be used to treat patients who are not suitable for taking surgery.^8^

Small nucleolar RNA host gene 16 (SNHG16) belongs to long noncoding RNA (lncRNA) family, and has been proved to be associated with the tumorigenesis of many cancer types,^9^, ^10^ moreover, lncRNAs act frequently as a tumor suppressor or promoter of the activation of oncogene.^11^, ^12^ SNHG16 gene was reported to be related to a series of diseases. Through competitively targeting miR‐146a‐5a with CCL5, SNHG16 could aggravate acute pneumonia by lowering its cell viability and promoting the apoptosis.^13^ SNHG16 was also widely considered as playing an important role in the oncogenic functions. In pancreatic cancer, SNHG16 was found to stimulate tumor growth via competitive sponging with miR‐218‐5p to regulate its direct target HMGB1.^14^ Moreover, SNHG16 have high expression in glioma, and it could function with EGRF through moderating miR‐373‐3p to activate PI3K/AKT signaling pathway, thus acting as an oncogene.^15^

MicroRNAs (MiRNAs) are a family of small noncoding RNA molecules, and have the ability of regulating gene expression in many diseases.^16^, ^17^ Furthermore, the oncogenic function or tumor suppression ability of miRNA in moderating a wide range of intercellurlar processes from cell proliferation to migration and invasion suggest that it is possibly involved in tumorigenesis of a variety of cancers. MiR‐23b‐3p, a family member belonging to miRNA has been reported to play a critical role in many tumors. Through suppressing PGC1α, miR‐23b‐3p was able to moderate cell metabolism by promoting cell proliferation.^18^ Also, the level of miR‐23b‐3p in serum also showed that miR‐23b‐3p could be a biomarker in the diagnosis of early HCC.^19^

In this study, the role of lncRNA SNHG16 in the regulation of autophagy of sorafinb‐resistant Hep3B cells was investigated, and its further regulation of miR‐23b‐3p and its downstream gene EGR1 was explored.

## 2 MATERIALS AND METHODS

### 2.1 Cells and tissues

The Hep3B cells were purchased from American Type Culture Collection (ATCC HB‐8064, ACTT) and cultured in ATCC‐formulated Eagle's minimum essential medium (30‐2003, ATCC) with 10% FBS.

A total of 40 human HCC with sorafenib resistance and its adjacent tissue, and 40 normal human HCC and its adjacent tissues were clinically collected from May 01, 2008 to June 30, 2018. Informed consent form was signed by each patient, and the study was approved by the Ethics Committee (2016). Drug resistance standard: patients who take sorafenib initially are considered to be ineffective, or the disease progresses after taking the drug for a period of time, and the increased dose is ineffective, and the disease worsens in a short time, and death is considered to be the occurrence of sorafenib resistance. Otherwise, sorafenib is considered sensitive.

### 2.2 Construction of sorafenib resistant in Hep3B cells

Hep3B cells were exposed to different doses of sorafenib to develop drug resistance in Hep3B. The cells were first cultured in a low dose (0.625 µmol/L) of sorafenib to allow them to stably grow for 2 weeks, and then the medium was replaced by normal medium for adjustment for 2 weeks. Next, the cells were continually incubated in 1.25 µmol/L sorafenib for approximately 2 weeks, and then in normal medium for 2 weeks. The circulation was repeated until the sorafenib dose reach 10 µmol/L, and the sorafenib‐resistant cells (Hep3/So) were obtained. After the establishment, the cell resistance of sorafenib was strengthened by constant culture with the sorafenib. After the cell model had been established, the cell morphology was observed under and inverted microscope and photographed.

### 2.3 Microarray‐based gene expression analysis

Total RNAs from Hep3B and Hep3B/So were harvested by TRIzol regent (Invitrogen), then gene expression profiling was processed by Axon GenePix 4000B (AXON) and read by GenePix pro V6.0 (AXON). Human LncRNA Microaray V1.0 (Arraystar) was used to analyze the data of five replicate samples, calculate the *p*‐value of the gene expression difference and statistical significance, and use the Fold Change (FC) ≥1.5 or ≤−1.5, *P* < .05 for t test as a criterion for differential gene screening. And the data are shown in Table 1.

**TABLE 1:** The lncRNA microarray data from Hep3B and Hep3B/So cells

lncRNA name	Fold change	*P*‐value	Regulation	Chromosome location
linc‐MAN1A2‐2	2.12	.005	Up‐regulation	Chr1
linc‐ENOSF1	2.69	.011	Up‐regulation	Chr18
linc‐SNHG16	15.66	.016	Up‐regulation	Chr17
linc‐NXPH2‐2	1.85	.018	Up‐regulation	Chr2
linc‐NFIA‐1	1.63	.022	Up‐regulation	Chr1
linc‐TYRP1‐7	1.6	.023	Up‐regulation	Chr9
linc‐AMPD2	1.59	.026	Up‐regulation	Chr1
linc‐ZNF322A‐4	1.57	.026	Up‐regulation	Chr6
linc‐SHOX‐4	1.52	.028	Up‐regulation	ChrX
linc‐SLITRK1‐6	2.7	.030	Down‐regulation	Chr13
linc‐MAGEA1‐1	2.03	.032	Down‐regulation	ChrX
linc‐MYO3A‐1	2.38	.033	Down‐regulation	Chr10
linc‐SMC1B‐3	2.17	.034	Down‐regulation	Chr22
linc‐GPR45‐2	2	.037	Down‐regulation	Chr2
linc‐ZFAT‐3	1.96	.040	Down‐regulation	Chr8
linc‐XBP1	1.81	.041	Down‐regulation	Chr22
linc‐MMD‐1	1.72	.042	Down‐regulation	Ch17
linc‐MERTK‐3	1.56	.046	Down‐regulation	Chr2

Note

Fold change: (−1.5, 1.5), *P* < .05.

### 2.4 Gene prediction and dual‐luciferase reporter assay

The target‐binding region of SNHG16 and miR‐23b‐3p was predicted by LncBase Predicted v.2 (http://carolina.imis.athena‐innovation.gr/diana_tools/web/index.php?r=lncbasev2%2Findex),^20^ and the threshold was set at score >0.9.

The wild‐type 3’ UTR of SNHG16 segment containing miR‐23b‐3p site was cloned and inserted into pmirGLO vector (Progema), whereas the mutant 3’UTR was constructed and inserted into pcDNA 3.1 vector (ThermoFisher). Similarly, the 3’ UTR of EGR1 with putative miR‐23b‐3p‐binding site segment was constructed and inserted into pmirGLO vector (Promega). The cells were cultured in a 96‐well plates, the vectors obtained above, equal amount of empty vectors, miR‐23b‐3p and its negative control were, respectively, transfected into Hep3B or Hep3B/So cells treated by Lipofectamine® 2000 (Invitrogen). Twenty‐four hours after the transfection, the luciferase activity was measured by dual luciferase reporter assay system (Promega). The Renilla luciferase activity served as reference.

### 2.5 CCK‐8 assay

The IC50 of normal Hep3B and sorafenib‐resistant Hep3B cells was detected by CCK‐8 assay. Sorafenib at the concentrations of 1, 2, 4, 8, 16, and 32 μmol/L were used to pretreat Hep3B or Hep3B/So cells for 24 hours, and the cell viabilities were detected. The effect of SNHG16 or miR‐23b‐3p on sorafenib resistance on Hp3B cells was determined by CCK‐8 assay. The cells were cultured in a 96‐well plates at 1.5 × 10^4^ cells per well. According to the protocol of Cell Counting Kit‐8 (HY‐K0301; MedChemExpress), the cells were suspended in 10 µL CCK8 solution for 3 hours at room temperature, and the absorbance was measured at the wavelength of 450 nm using a microplate reader (Bio‐Rad 680).

### 2.6 Flow cytometry

The apoptosis rates of control, transfected Hep3B, or Hep3B/So cells were determined by Annexin V‐FITC Early Apoptosis Detection Kit (#6592; Cell Signaling). Hep3B cells were suspended in 500 µL binding buffer and then incubated at room temperature. 10 µL Annexin V‐FITC and 5 µL PI was added into the cells and further incubated in the dark. Then, cell apoptosis rate was measured using Attune NxT Flow Cytometer (attune Nxt Software version 2.5, 2019; ThermoFisher).

### 2.7 Cell transfection

The amplification of SNHG16 was constructed from cDNA using primer sequences forward 5ʹ‐CCCAAGCTTGCGTTCTTTTCGAGGTCGGC‐3ʹ, and reverse 5ʹ‐CCGGAATTCTGACGGTAGTTTCCCAAGTTTATTGTAAGT‐3ʹ, then pcDNA3.1‐SNHG16 (Invitrogen) and its control vector were synthesized. SiSNHG16 and negative control were purchased from Sigma‐Aldrich, and MiR‐23b‐3p inhibitor (miR20000418‐1‐5) and negative control were obtained from Rib‐Bio. The Lipofectamine 2000 (Invitrogen) was used for Hep3B and Hep3B/So cell transfection following the manufacturer's protocol. After the transfection, the Hep3B cells were divided into control, pc‐control or si‐control, pc‐control or si‐SNHG16, with two subgroups in each group, one group of cells was treated by 3.14 μmol/L sorafenib, whereas the other was treated by equal amount of solvent. Hep3B/So cells were similarly treated.

### 2.8 Reverse transcription quantitative PCR

The expressions of SNHG16, EGR1, and miR‐23b‐3p were determined by qRT‐PCR. The total RNA was harvested from control or transfected Hep3B or Hep3B/So cells using TRIzol reagent (ThermoFisher) and the concentration was determined by NanoDrop2000c (ND‐2000, ThermoFisher). For SNHG16 and EGR1, the transcription from RNA to cDNA was conducted by Prime Script RT Reagent Kit (Takara), and for miR‐23b‐3p, the transcription was conducted by Prime‐Script miRNA cDNA Synthesis Kit (TaKaRa). Step One Plus Real‐Time PCR system (Applied Biosystems) was performed under the following conditions: at 95°C for 20 minutes, then 40 cycles at 94°C for 15 seconds, at 55°C for 30 seconds and finally at 72°C for 30 seconds. The relative quantification analysis was performed by 2−^△△CT^ method. GAPHD and U6 served as internal reference for SNHG16 and EGR1, miR‐23b‐3p, respectively. The sequences of primers used in this study are shown in Table 2.

**TABLE 2:** Primers sequence used for qPCR

Primer	Sequence
SNHG16	
Forward	5ʹ‐CAGAATGCCATGGTTTCCCC‐3ʹ
Reverse	5ʹ‐TGGCAAGAGACTTCCTGAGG‐3ʹ
miR‐23b‐3p	
Forward	5ʹ‐CAGGCAAGATGCTGTTGCA‐3ʹ
Reverse	5ʹ‐GCGAGCACAGAATTAATACGACTC‐3ʹ
EGR1	
Forward	5ʹ‐TGACCGCAGAGTCTTTTCCT‐3ʹ
Reverse	5ʹ‐TGGGTTGGTCATGCTCACTA‐3ʹ
U6	
Forward	5ʹ‐CTCGCTTCGGCAGCACA‐3ʹ
Reverse	5ʹ‐AACGCTTCACGAATTTGCGT‐3ʹ
GADPH	
Forward	5ʹ‐CGGAGTCAACGGATTTGGTCGTAT‐3ʹ
Reverse	5ʹ‐AGCCTTCTCCATGGTGGTGAAGAC‐3ʹ

### 2.9 Western blot

Cell lysate derived from Hep3B or Hep3B/So was prepared using lysis buffer (PBS + 1% NP40 + 0.1% SDS + 5 mmol/L EDTA + 0.5% sodium deoxycholate + 1 mmol/L sodium orthovanadate), and protein concentration was determined by BCA Assay Kit (ThermoScientific). 40 μg protein samples were separated on 10% SDS‐PAGE and then electrotransferred onto PVDF membrane, which was blocked by 5% skimmed milk at room temperature for 1 hour Next, the membrane was incubated with the primary antibodies LC3I (GTX17380, 1:1000; GeneTex) and LC3II (GTX127375, 1:3000; GeneTex), EGR1 (ab133695, 1:1000; Abcam) at 4°C overnight. The membrane was rinsed in TBST solution and then incubated with the secondary goat anti‐rabbit antibody (ab205718, 1:5000; Abcam) for 2 hours. The signal was determined using enhanced ECL (Perkin Elmer) by ImageQuant LAS 4000 (ImageQuantTL, Imagemoster 2D Platinum 6.0; GE Healthcare Life Science).

### 2.10 NOD/SCID mice model establishment and tumorigenesis assay

The animal experiment was conducted following the Guide for the Care and Use of Laboratory Animals and approved by the Ethics Committee. Thirty‐five 4‐week‐old NOD/SCID male mice with similar weight were obtained (Vital River) and subcutaneously injected with 200 μL 5 × 10^6^ Hep3B, Hep3B/So, Hep3B/So + si‐SNHG16, Hep3B/So + inhibitor, and Hep3B/So + si‐SNHG16 + inhibitor cells, respectively, to the right flank area. The mice were fed in an isolator at 24 ± 2°C under 12‐hour lighting cycle, the ventilation was maintained 20 times per hour, and the mice were provided with free access to food and tap water. The tumors were closely monitored until it became obvious, and then the tumor size was recorded every 3 days and calculated according to the equation: 0.52 × length. When tumor size exceeded approximately 200 mm^3^, the mice injected with Hep3B and Hep3B/So cells were daily injected with 100 mg/kg solvent once a week as control group, whereas other mice injected with Hep3B, Hep3B/So, Hep3B/So + si‐SNHG16, Hep3B/So + inhibitor, and Hep3B/So + si‐SNHG16 + inhibitor cells were injected with 100 mg/kg sorafenib. 3 weeks later, the mice were sacrificed and tumor was separated from mice, tumor protein was harvested and EGR1 expression was determined by qRT‐PCR and western blot, LC3II and LC3I expressions were detected by western blot.

### 2.11 Statistical analysis

The data were shown as the means ± standard deviation. SPSS 17.0 was used for analysis. Multiple comparisons were accessed by one‐way ANOVA, followed by Dunnett's post hoc test. Student’s t‐test was used for comparing the difference between two groups. The correlation analysis between SNHG16 and miR‐23b‐3p was determined by *Person* correlation analysis. *P* < .05 was considered as statistically significant difference. The experiments were repeated in triplicate.

### 2.12 Public database

Public TCGA (https://portal.gdc.cancer.gov/) database for hepatocellular carcinoma (HCC) was used for survival analysis. The Kaplan‐Meier method was applied for the comparison between the groups.

## 3 RESULT

### 3.1 Cell morphology, gene expression, and sorafenib resistance between Hep3B and Hep3B/So cells

**FIGURE 1:**
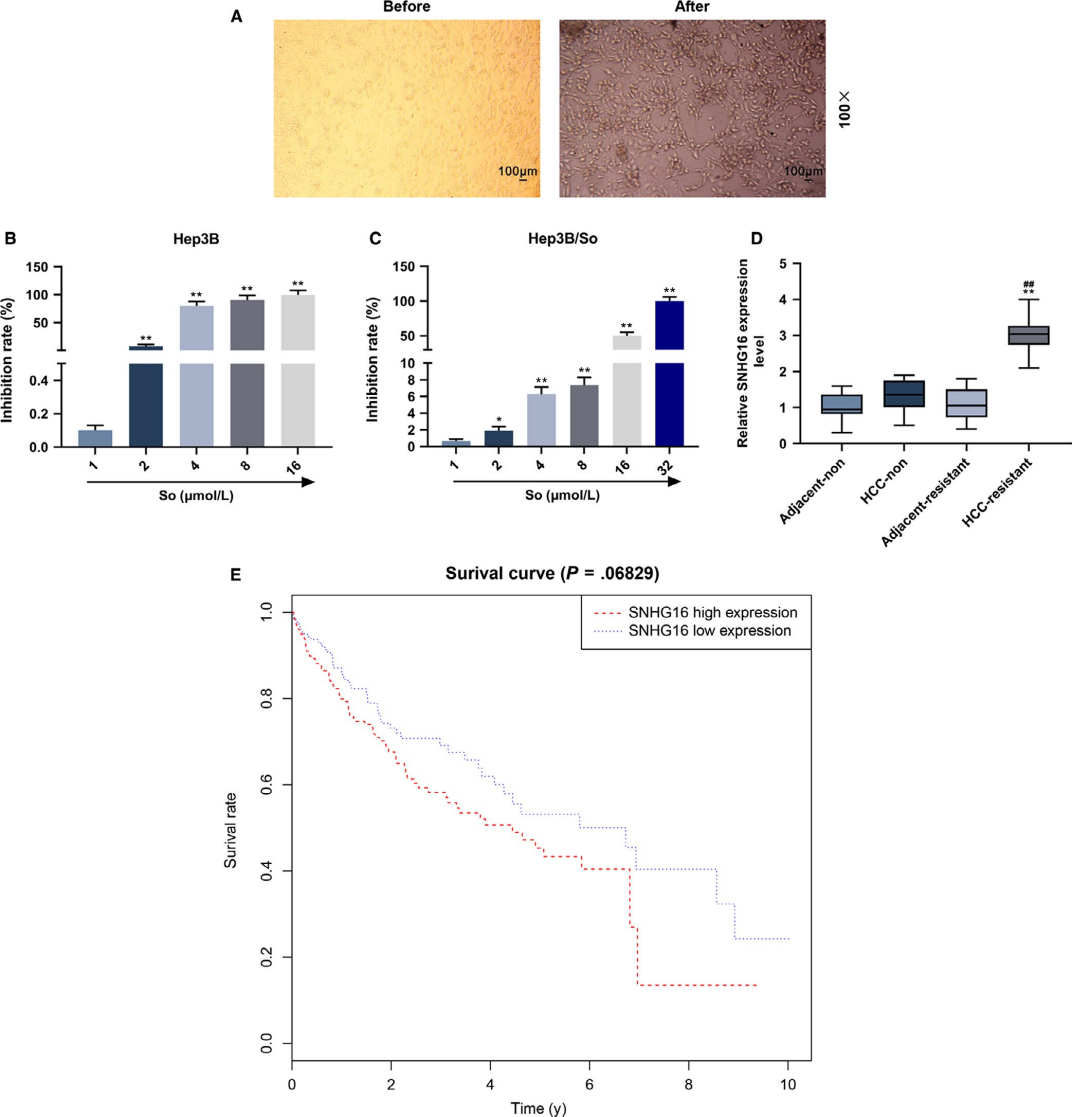
Cell morphology and sorafenib resistance detection between Hep3B and Hep3B/So cells and SNHG16 expression. A, Morphology of Hep3B and Hep3B/So cells observed under inverted mircorscope. B, C, Cell viability of Hep3B and Hep3B/So cells under different doses of sorafenib determined by CCK‐8 assay (Sorafenib dose for Hep3B: 1, 2, 4, 8, 16 μmol/L, and Sorafenib dose for Hep3B/So: 1, 2, 4, 8, 16 and 32 μmol/L). *P < .05, **P < .001, vs 1 μmol/L. D, SNHG16 expression in normal and sorafenib‐resistant tissues and their adjacent tissues detected by qTR‐PCR, the group was divided into Adjacent‐non, HCC‐non, Adjacent‐resistant and HCC‐resistant, GAPDH served as internal reference. E, Survival analysis based on TCGA of SNHG16 expression. *P < .05, **P < .001, vs HCC‐non. ^##^ *P* < .001, vs Adjacent‐resistant

The sorafenib‐resistant Hep3B cell model were constructed by increasing sofafibnib dose and the cell morphology was observed (Figure 1A). Normal Hep3B cells mainly had a round‐like shape and closely attached to each other, whereas most of Hep3B/So cells present fusiform or lobular, with euchromatin predominating in the nucleus and the formation of pseudopod, and distance between cells became larger. Then microarray was used to investigate the gene expression changes after sorafenib resistance was obtained in Hep3B cells. As shown in Table 1, SNHG16 expression significantly increased, the level was five times more than the gene with the second highest expression level. The sorafenib resistance in Hep3B and Hep3B/So groups was then detected (Figure 1B,C), and we found that the IC50 of Hep3B was 3.14 μmol/L, whereas Hep3B/So was 15.80 μmol/L, suggesting that Hep3B/So had a higher sorafenib resistance.

### 3.2 Expression of SNHG16 and survival analysis in sorafinib‐resistant and normal HCC tissues and their adjacent tissues

The expressions of SNHG16 in normal and sorafenib‐resistant HCC tissues and their adjacent tissues were determined by qRT‐PCR (Figure 1D), and we found that the SNHG16 expression was the highest in HCC tissues with sorafenib resistance compared with other cells. According to survival analysis (Figure 1E), low expression of SNHG16 was not associated with better HCC prognosis and higher survival rate (*P* > .05).

### 3.3 The effect of SNHG16 on cell viability, apoptosis, and autophagy in normal and sorafenib‐resistant Hep3B cells

**FIGURE 2:**
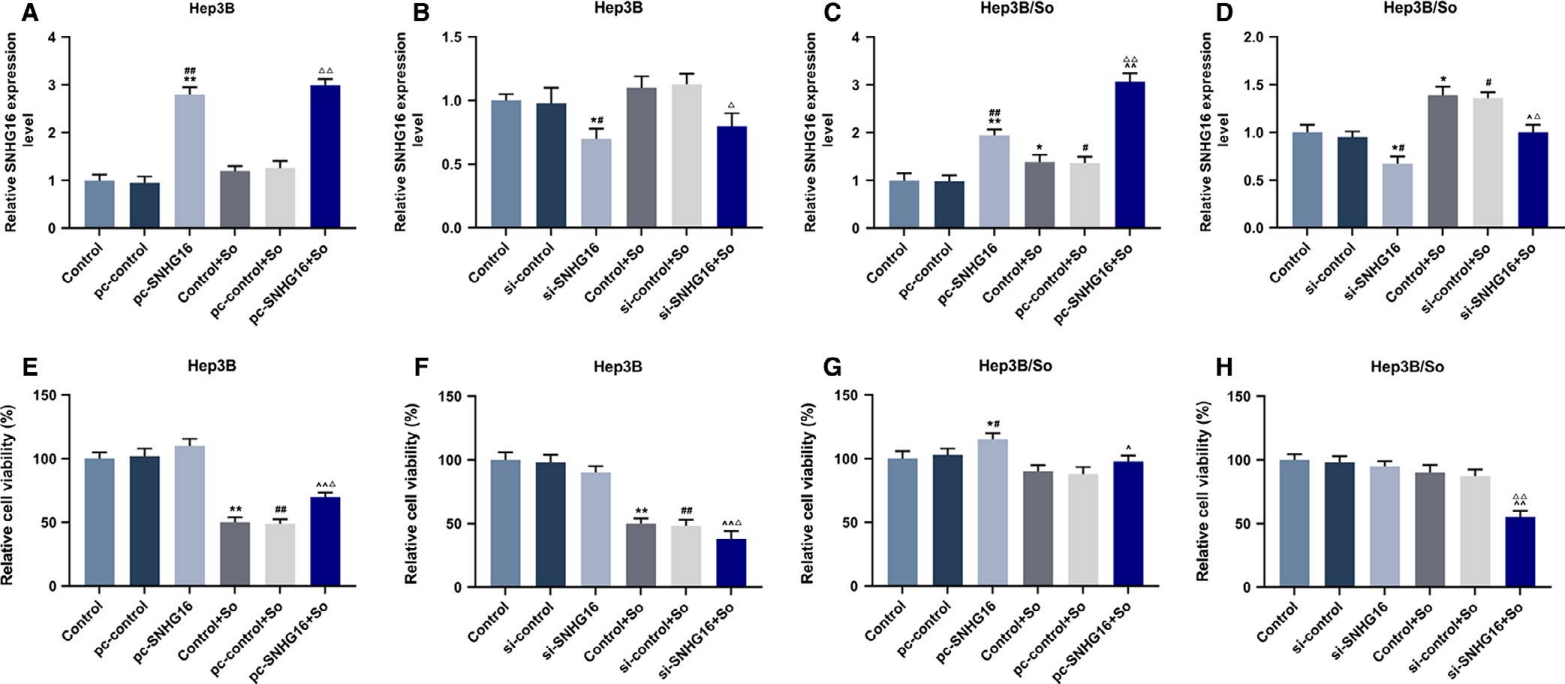
SNHG16 expression and cell viability in Hep3B or Hep3B/So cells, and the group was divided into control, pc‐control or si‐control, pc‐SNHG16 or si‐control, control + So, pc‐control + So or si‐control + So, pc‐SNHG16 + So or si‐SNHG16 + So. A‐D, The expression level of SNHG16 in Hep3B or Hep3B/So cells determined by qRT‐PCR, GAPDH served as internal reference. E‐H, Cell viability of Hep3B or Hep3B/So cells determined by CCK‐8. *P < .05, **P < .001, vs control. ^#^ *P* < .05, ^##^ *P* < .001, vs pc‐control or si‐control. ^^^ *P* < .05, ^^^^ *P* < .001, vs pc‐control + So or si‐control + So, ^△^ *P* < .05, ^△△^ *P* < .001, vs pc‐control + So or si‐control + So

**FIGURE 3:**
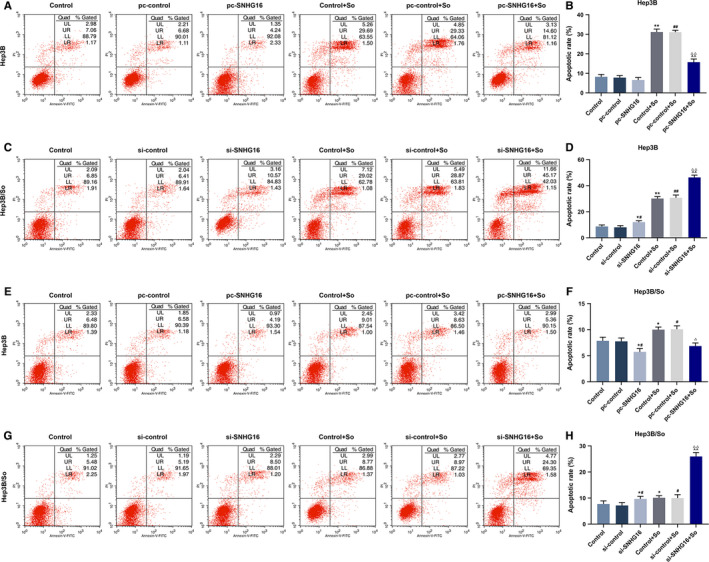
Cell apoptosis in Hep3B or Hep3B/So cells, with the group divided into control, pc‐control or si‐control, pc‐SNHG16 or si‐control, control + So, pc‐control + So or si‐control + So, pc‐SNHG16 + So or si‐SNHG16 + So. A‐D, Cell apoptosis in Hep3B cells determined by flow cytometry. E‐H, Cell apoptosis in Hep3B/So cells determined by flow cytometry. *P < .05, **P < .001, vs control. ^#^ *P* < .05, ^##^ *P* < .001, vs pc‐control or si‐control. ^^^^ *P* < .001, vs pc‐control + So or si‐control + So, ^△^ *P* < .05, ^△△^ *P* < .001, vs pc‐control + So or si‐control + So

**FIGURE 4:**
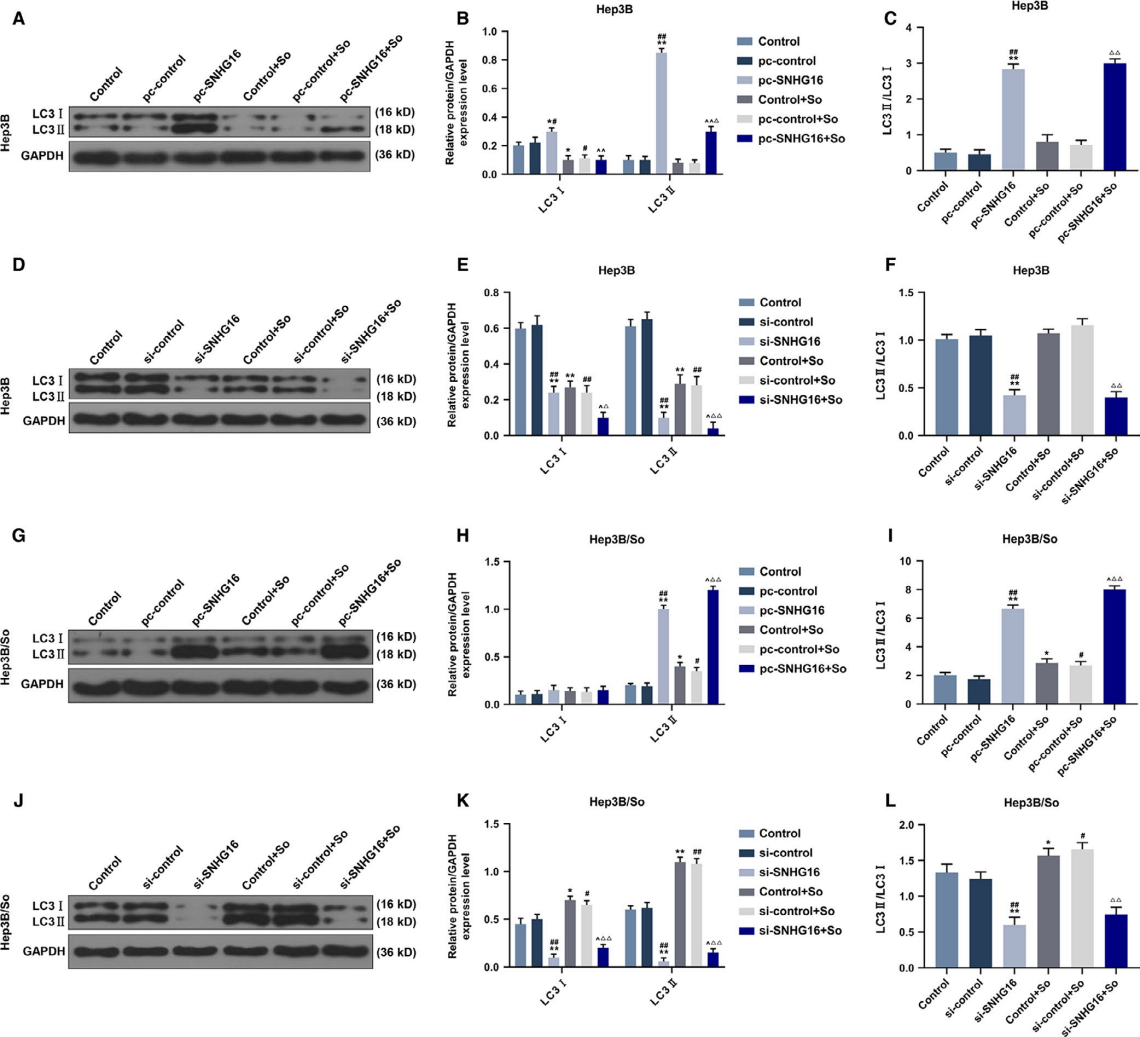
The expression levels of LC3II and LC3 and the ratio of LC3II and LC3I in Hep3B or Hep3B/So cells, with the group divided into control, pc‐control or si‐control, pc‐SNHG16 or si‐control, control + So, pc‐control + So or si‐control + So, pc‐SNHG16 + So or si‐SNHG16 + So, GAPDH served as internal reference. A‐D, LC3II and LC3I and LC3II/LC3I in Hep3B cells determined by western blot. E‐H, LC3II and LC3I and LC3II/LC3I in Hep3B cells determined by western blot. *P < .05, **P < .001, vs control. ^#^ *P* < .05, ^##^ *P* < .001, vs pc‐control or si‐control. ^^^ *P* < .05, ^^^^ *P* < .001, vs pc‐control + So or si‐control + So, ^△^ *P* < .05, ^△△^ *P* < .001, vs pc‐control + So or si‐control + So

**FIGURE 5:**
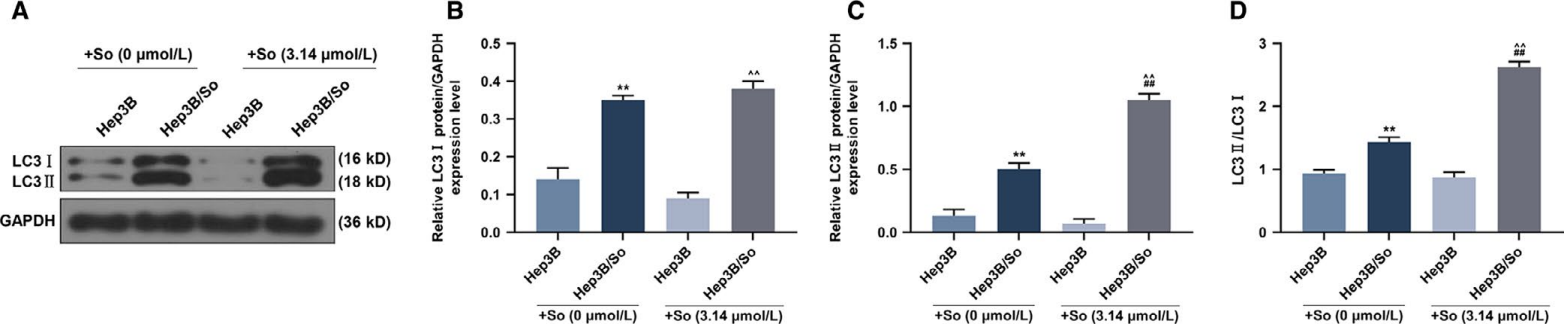
Comparison of LC3II and LC3 expressions in Hep3B or Hep3B/So cells under the same sorafenib dose. A‐C, The expression levels of LC3II and LC3I at a sorafenib dose of 0 and 3.14 μmol/L. D, Ratio of LC3II and LC3I at a sorafenib dose of 0 and 3.14 μmol/L. **P < .001, vs Hep3B + So (0 μmol/L), ^##^ *P* < .001, vs Hep3B/So + So (0 μmol/L), ^^^^ *P* < .001, vs Hep3B + So (3.14 μmol/L)

SNHG16 expression level of transfected Hep3B and Hep3B/So cells and their control cells were determined by qRT‐PCR (Figure 2A‐D). As we observed, the SNHG16 expression level was high when the gene was overexpressed, similarly, low SNHG16 expression level was detected when the gene expression was silenced. Then cell viability in those cell groups was detected (Figure 2E‐H). In Hep3B cells, the up or down‐regulation of SNHG16 without sorafenib had no significant effect on cell viability, however, when the cells were treated by sorafenib, the up‐regulation of SNHG16 could promote the cell viability, whereas the inhibition of SNHG16 expression tended to suppress cell viability. Moreover, sorafenib could suppress cell viability. Similar results were shown in Hep3B/So cells, as the overexpression of SNHG16 was promoted cell viability, whereas the down‐regulation of SNHG16 inhibited cell viability. Furthermore, increasing the concentration of sorafenib suppressed the viability of Hep3B/So cells as well. Cell apoptosis was then determined (Figure 3), we found that the sorafenib treatment significantly increase the cell apoptosis rate. In Hep3B cells, the only up‐regulation of SNHG16 expression had no significant effect on cell apoptosis, whereas the combination of overexpressed SNHG16 and sorafenib treatment significantly suppressed the cell apoptosis rate compared with the group only treated with sorafenib, on the other hand, silencing SNHG16 was found to increase the cell apoptosis rate. In Hep3B/So cells, the up‐regulation of SNHG16 was able to inhibit the cell apoptosis, whereas the down‐regulation of SNHG16 was shown to promote cell apoptosis. Moreover, the LC3II and LC3I expressions were determined by western blotting (Figure 4), in Hep3B cells, the effect of sorafenib on LC3II/LC3I protein expression level was not significant, however, the up‐regulation of SNHG16 significantly up‐regulated the LC3II/LC3I level, which was greatly down‐regulated by SNHG16 (*P* < .001). In Hep3B/So cells, sorafenib could increase the value of LC3II/LC3I, and similarly, up‐regulation of SNHG16 was found to significantly elevate LC3II/LC3I level, which could be greatly down‐regulated by SNHG16. Moreover, the LC3II/LC3I expression level between Hep3B and Hep3B/So was compared (Figure 5), and results showed that the level of LC3II/LC3 was significantly higher in Hep3B/So cells than that in Hep3B cells, suggesting that he treatment of sorafenib was able to further up‐regulate LC3II/LC3I level.

### 3.4 SNHG16 targeted miR‐23b‐3p to affect viability, apoptosis, and LC3II/LCI expression

**FIGURE 6:**
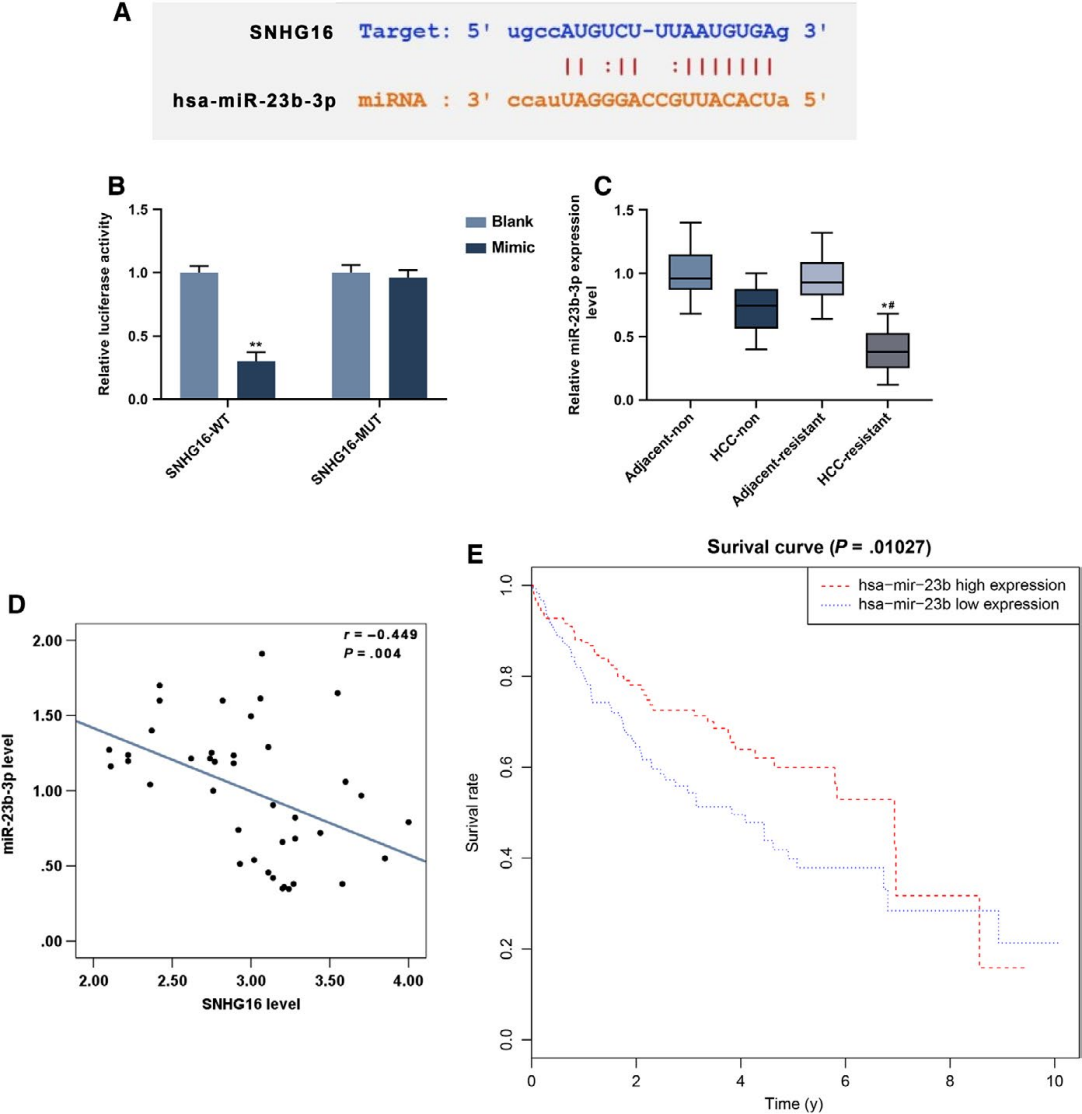
Prediction and verification of SNHG16 target gene. A, Prediction of SNHG16 target gene. B, Verification of SNHG16 target gene detected by dual‐luciferase reporter assay. *P < .05, **P < .001, vs Mimic. C, The expression of miR‐23b‐3p in normal and sorafenib‐resistant HCC tissues determined by qRT‐PCR, GAPDH served as internal reference. D, Correlation analysis between SNHG16 and miR‐23b‐3p expression in normal and sorafenib‐resistant tissues and their adjacent tissues. E, Survival analysis based on TCGA of miR‐23b‐3p expression. *P < .05, **P < .001, vs HCC‐non. ^#^ *P* < .05, vs Adjacent‐resistant

**FIGURE 7:**
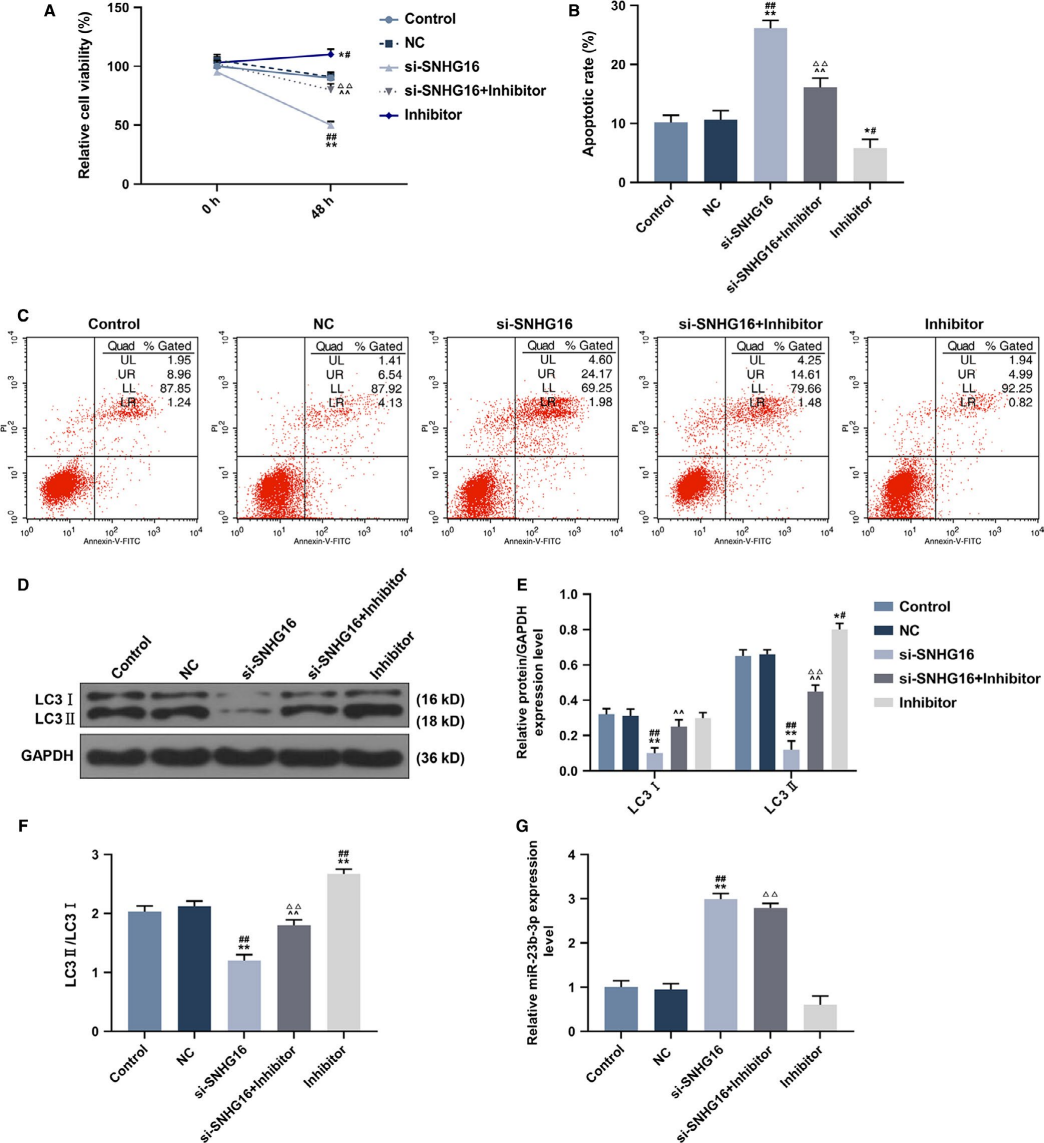
The correspondence between SNHG16 and miR‐23b‐3p in Hep3B/So cells, with the group divided into control, NC, si‐SNHG16, si‐SNHG16 + inhibitor, inhibitor. A, Cell viability detected by CCK‐8 assay at 0 and 48 h. B‐C, Cell apoptosis detected by flow cytometry. D‐F, The expression of LC3II and LC3I and the ratio of LC3II/LC3I detected by western blot. G, miR‐23b‐3p expression level determined by qRT‐PCR. *P < .05, **P < .001, vs control, ^#^ *P* < .05, ^##^ *P* < .001, vs NC, ^^^^ *P* < .001, vs si‐SNHG16, ^△△^ *P* < .001, vs Inhibitor

The target gene of SNHG16 was predicted to be miR‐23b‐3p (Figure 6A), and further verified (Figure 6B). Compared with other three groups, the SNHG16‐WT had the lowest luciferase activity (*P* < .001), which proved the relationship between SNHG16 and miR‐23b‐3p. Meanwhile, the expression of miR‐23b‐3p in normal and sorafenib resistance tissues and their adjacent tissues was determined. In HCC‐resistance tissues, the miR‐23b‐3p expression was the lowest (Figure 6C), and a negative correction between miR‐23b‐3p and SNHG16 expression in sorafenib‐resistant HCC tissues was observed (Figure 6D). Nevertheless, the survival analysis showed that high miR‐23b‐3p expression was related to high survival rate (Figure 6E). The effect of miR23b‐3p inhibitor on cell viability was explored (Figure 7A), and we observed that the down‐regulation of SNHG16 dramatically inhibited cell viability (*P* < .001), whereas miR‐23b‐3p inhibitor significantly promoted the cell viability (*P* < .001). Then cell apoptosis was then detected, as shown in Figure 7B,C, the silence of SNHG16 significantly increased cell apoptosis rate (*P* < .001), whereas the transfection of miR‐23b‐3p inhibitor greatly lower the cell apoptosis rate (*P* < .001). The expressions of LC3 and LC3I were also been determined, Figure 7D‐F showed that the silence of SNHG16 significantly lowered the ratio of LC3II and LC3I expression levels (*P* < .001), whereas the si‐SNGH16 + miR‐23b‐3p inhibitor was found to greatly increase the LC3II/LC3I ratio (*P* < .001). The determination of miR‐23b‐3p expression was determined (Figure 7G), and the results showed that the silence of SNHG16 significantly up‐regulated the expression of miR‐23b‐3p (*P* < .001), which was greatly lowed by the miR‐23b‐3p inhibitor (*P* < .001).

### 3.5 MiR‐23b‐3p suppressed high EGR1 expression in sorafenib‐resistant tissues

**FIGURE 8:**
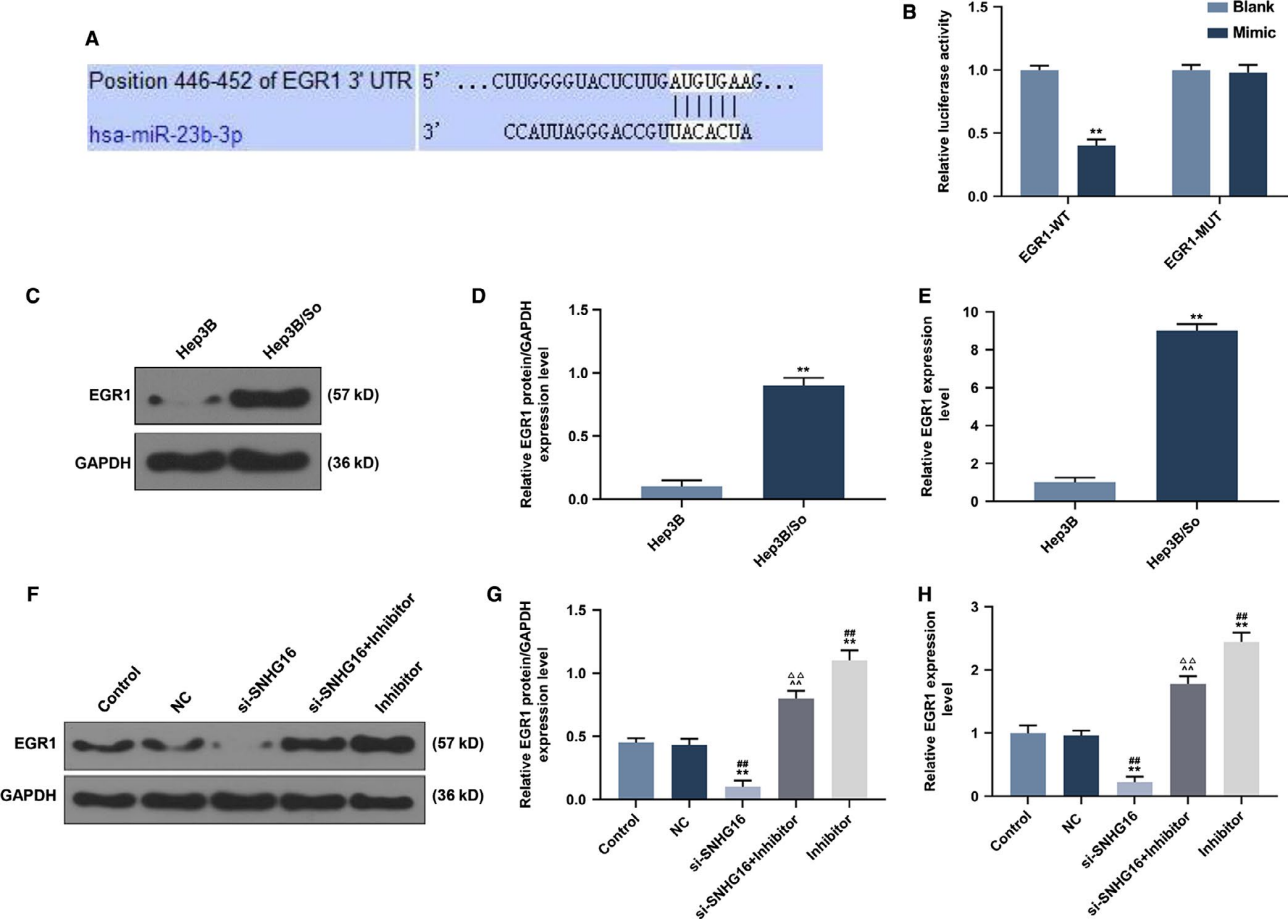
Prediction and verification of miR‐23b‐3p target gene and the expression of EGR1 in Hep3B or Hep3B/So cells. A, Prediction of miR‐23b‐3p target gene. B, Verification of miR‐23b‐3p target gene detected by dual‐luciferase reporter assay. *P < .05, **P < .001, vs Mimic. C‐E, EGR1 protein and gene expression in Hep3B and Hep3B/So cells determined by qRT‐PCR, GAPDH served as internal reference. *P < .05, **P < .001, vs Hep3B. F‐H, EGR1 protein and gene expressions in Hep3B/So cells detected by qRT‐PCR, with the group divided into control, NC, si‐SNHG16, si‐SNHG16 + inhibitor and inhibitor, GAPDH served as internal reference. **P < .001, vs control, ^##^ *P* < .001, vs NC, ^^^^ *P* < .001, vs si‐SNHG16, ^△△^ *P* < .001, vs Inhibitor

The target gene for miR‐23b‐3p was predicted (Figure 8A) to be EGR1 and further verified (Figure 8B), as the significantly low luciferase activity in EGR1‐WT group confirmed that EGR1 was the target gene for miR‐23b‐3p. Then the EGR1 expression in Hep3B and Hep3B/So cells was determined (Figure 8C,D), we observed that both gene and protein expressions of EGR1 in Hep3B/So were significantly higher than those in Hep3B (Figure 8E, *P* < .001). Moreover, the effect of miR‐23b‐3p inhibitor on EGR1 protein and gene expressions in Hep3B/So cells was detected (Figure 8F‐H), and data showed that the silence of SNHG16 significantly lowered the gene and protein expressions of EGR1 (*P* < .001), whereas the inhibition of miR‐23b‐3p greatly increased the expression of EGR1 (*P* < .001).

### 3.6 Overexpression of EGR1 partially reversed the effects of overexpression of miR‐23b‐3p on cell viability, apoptosis and autophagy sorafenib‐resistant Hep3B cells

**FIGURE 9:**
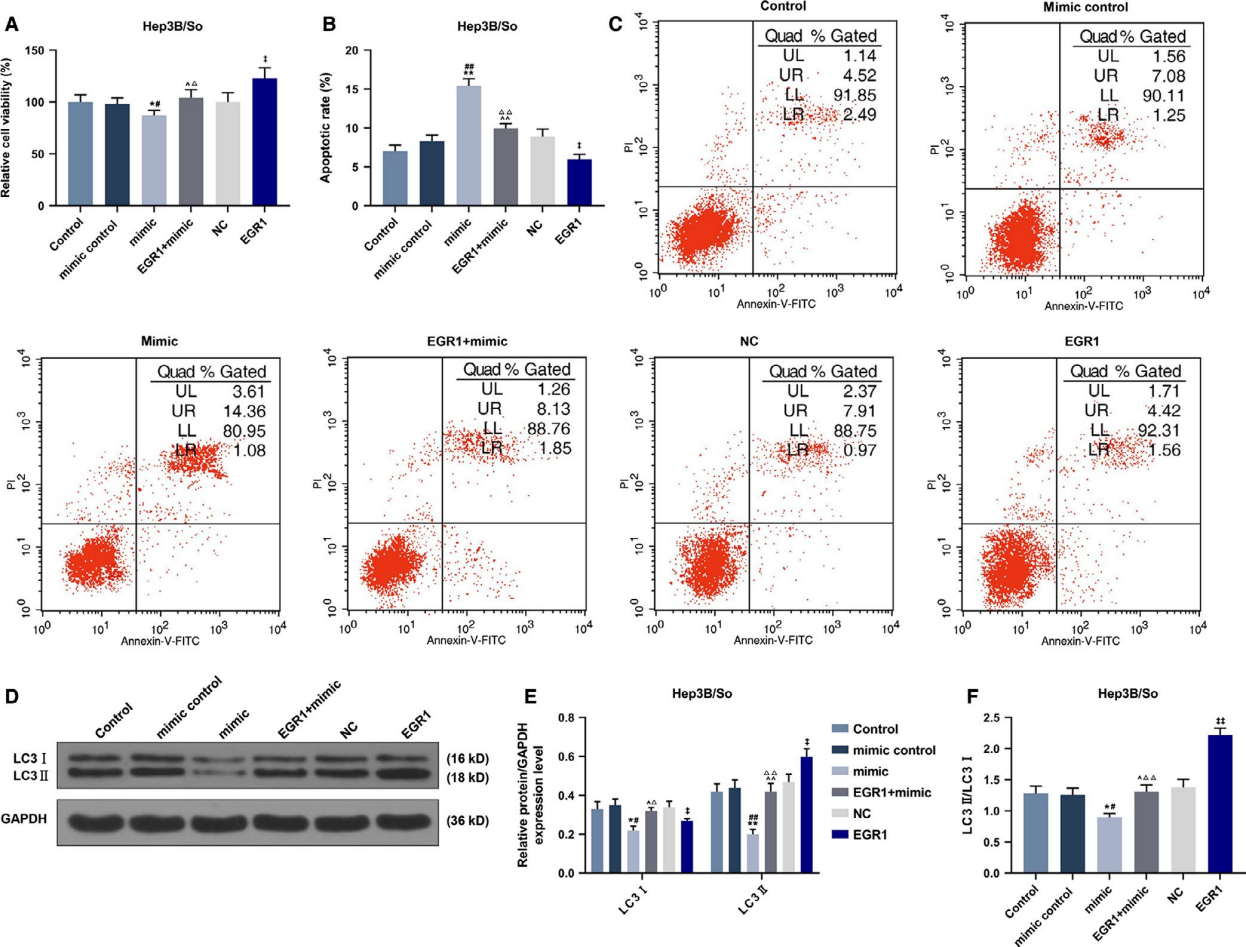
Overexpression of EGR1 partially reversed the effects of overexpression of miR‐23b‐3p on cell viability, apoptosis and autophagy sorafenib‐resistant Hep3B cells. A, CCK‐8 assay was used to detect the viability of sorafenib‐resistant Hep3B cells. B and C, Flow cytometry was used to detect the apoptosis of sorafenib‐resistant Hep3B cells. D‐F, Western blot was used to detect the expression of LC3I and LC3II in sorafenib‐resistant Hep3B cells. *P < .05, **P < .001, vs control, ^#^ *P* < .05, ^##^ *P* < .001, vs mimic control, ^^^ *P* < .05, ^^^^ *P* < .001, vs mimic, ^△^ *P* < .05, ^△△^ *P* < .001, vs EGR1, ^‡^ *P* < .05, ^‡‡^ *P* < .001, vs NC

EGR1 overexpression vector and miR‐23b‐3p overexpression vector were transfected into sorafenib‐resistant Hep3B cells, respectively, to explore the effects of EGR1 and miR‐23b‐3p on the drug resistance mechanism of cancer cells. As shown in Figure 9A, miR‐23b‐3p mimic inhibited the viability of sorafenib‐resistant Hep3B cells, but the overexpression of EGR1 could reverse the inhibitory effect of overexpressed miR‐23b‐3p on cell viability (*P* < .001). In addition, overexpressed miR‐23b‐3p promoted apoptosis of sorafenib‐resistant Hep3B cells, whereas the overexpression of EGR1 could reverse the promoted effect of overexpressed miR‐23b‐3p on cell apoptosis (*P* < .001, Figure 9B,C). Moreover, overexpressed miR‐23b‐3p inhibited LC3II protein expression, but overexpressed EGR1 could reverse the inhibitory effect of overexpressed miR‐23b‐3p on LC3II protein expression (*P* < .001, Figure 9D‐F).

### 3.7 Sorafenib resistance effect of SNHG16 and miR‐23b‐3p in Hep3B and Hep3B/So cells detected by Xenograft tumor model

**FIGURE 10:**
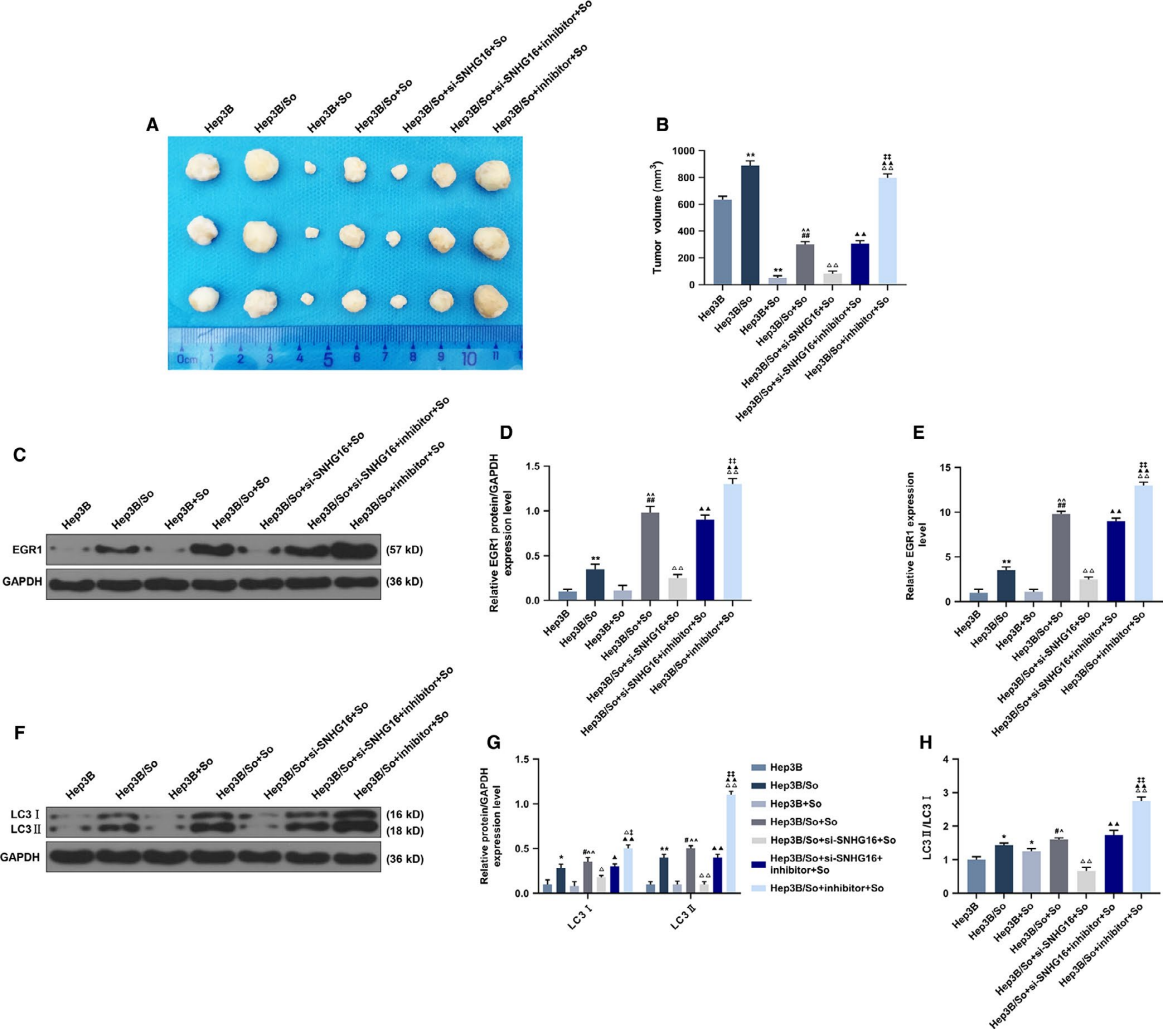
Tumorigenesis in mice injected with Hep3B or Hep3B/So cells and the expressions of EGR1 and LC3II and LC3I, with group divided into Hep3B, Hep3B/So, Hep3B + So, Hep3B/So + So, Hep3B/So + si‐SNHG16 + inhibitor+So, Hep3B/So + si‐SNHG16 + inhibitor+So, Hep3B/So + inhibitor+So. A and B, Tumor volume from scarified mice. C‐E, EGR gene and protein expression levels detected by qRT‐PCR and western blot. F‐H, LC3II, LC3I, and LC3II/LC3I expressions determined by western blot. ^*^ *P* < .05, ^**^ *P* < .001, vs Hep3B, ^#^ *P* < .05, ^##^ *P* < .001, vs Hep3B/So, ^^^ *P* < .05, ^^^^ *P* < .001, vs Hep3B + So, ^△^ *P* < .05, ^△△^ *P* < .001, vs Hep3B/So + So, ^▲^ *P* < .05, ^▲▲^ *P* < .001, vs Hep3B/So + si‐SNHG16 + So, ^‡^ *P* < .05, ^‡‡^ *P* < .001, vs Hep3B/So + si‐SNHG16 + inhibitor + So

The Hep3B or Hep3B/So cells transfected with different genes were injected into mice and the size of tumor was measured (Figure 10A,B). The treatment of sorafenib significantly inhibited the tumor growth of Hep3B and Hep3B/So, the effect on tumor size in Hep3B was significantly greater than which in Hep3B/So. The tumor size in group Hep3B/So + si‐SNHG16 + sorafenib was significantly smaller than in the group of Hep3B/So + sorafenib. Furthermore, miR‐23b‐3p in sorafenib‐treated Hep3B/So cells enlarged the tumor size more than Hep3B/So + So, Hep3B/So + si‐SNHG16 + So and Hep3B/So + si‐SNHG16 + inhibitor+So groups, suggesting that the inhibition of miR‐23b‐3p was able to significantly increase the tumor size. EGR1 expression was determined in tumor cells (Figure 10C‐E), sorafenib and si‐SNHG16 were found to significantly increase the EGR1 expression (*P* < .001), whereas the transfection of miR‐23b‐3p inhibitor significantly reduced the EGR1 expression (*P* < .001). Moreover, the protein expressions of LC3II and LC3I were measured, similarly, the treatment of sorafenib or silence of SNHG16 were observed to significantly increase the ratio of LC3II and LC3I, whereas the inhibition of miR‐23b‐3p was found to noticeably reduce the ratio (*P* < .001, Figure 10F‐H).

## 4 DISCUSSION

Studies showed that SNHG16 had a high expression in HCC cells under sorafenib resistance treatment.^21^ In this study, the further mechanism of the role of SNHG16 in the sorafenib‐resistant Hep3B cells was investigated, through sorafenib‐resistant Hep3B model, and we found that the resistance of sorafenib in Hep3B/So was nearly five times higher than that in Hep3B. The cell morphology change in Hep3B/So cells was observed, compared with normal Hep3B cells, the sorafenib‐resistant Hep3B cells was fusiform or lobular with loose structure. The microarray assessment found that SNHG16 expression was significantly high in Hep3B/So cells.

HCC has high mortality, and sorafenib is a multikinase inhibitor that is one of the few potent therapies for treating HCC. However, the sorafenib resistance acquired in HCC cells is the limitation of sorafenib in HCC treatment. In sorafenib‐resistant HCC cells, SNHG16 and some other genes such as FGF19, miR‐31‐5p were discovered to have high expression levels, and sorafenib induced HCC cells could elevated oxidative stress, then causing cell apoptosis.^22^, ^23^ Moreover, in both Hep3B and Hep3B/So cells, the overexpression of SNHG16 promoted cell viability, and partially reversed the viability inhibition caused by sorafenib treatment, whereas the silence of SNHG16 could suppress the cell viability. Similarly, overexpressed SNHG16 inhibited cell apoptosis, which partially compensate the adverse effect on cell apoptosis caused by sorafenib, whereas silence of SNHG16 promoted cell apoptosis. The cell autophagy levels of Hep3B and Hep3B/So cells were also examined, as the marker of autophagy, the ratio of LC3II and LC3I is often used to evaluate the autophagy level.^24^ In the progression of tumors, autophagy is a critical process for tumor cells to gain drug resistance and promote their proliferation ability. For example, the activation of ERK/MAPK signaling pathway promotes cell autophagy level, as a result, the resistance to cisplatin in ovarian cancer cells will be increased.^25^ In this study, the effect of sorafenib treatment on increasing cell autophagy in Hep3B/So cells was greater than that in Hep3B cells, moreover, the overexpression of SNHG16 elevated cell autophagy level, which, however, could be reduced by suppression of SNHG16. Noticeably, Hep3B/So had a higher autophagy level than Hep3B cells.

In sorafenib‐resistant HCC tissues, the expression of SNHG16 was up‐regulated, whereas miR‐23b‐3p was down‐regulated. SNHG16 was reported to alleviate the hydrogen peroxide‐induced injury in PC‐12 cells via up‐regulating miR‐423‐5p,^26^ and it could induce sorafenib resistance in HCC cells via moderating miR‐140‐5p.^27^ Moreover, SNHG16 was found to promote EMT process in bladder cancer via directly interacting with miR‐17‐5p,^28^ and it miR‐23b‐3p was found to be moderated by LncRNA HOTAIR to enhance the EMT process, resulting in acceleration of malignant HCC development.^29^ In this study, the survival analysis was conducted, and the results indicated that a poor prognosis was correlated with SNHG16 and miR‐23b‐3p expressions. As reported by He, the down‐regulation of miR‐23b‐3p expression was found to be a potential biomarker of HCC progression through TCGA survival analysis.^30^ However, there is no research providing any finding on the relationship between SNHG16 and miR‐23b‐3p. In this study, miR‐23b‐3p was predicted as the target gene for SNHG16, and the miR‐23b‐3p inhibitor was observed to partially reverse the effect of SNHG16 silence on inhibiting cell viability and autophagy, promote apoptosis, and elevate miR‐23b‐3p expression, suggesting that SNHG16 was associated with miR‐23b‐3p.

Early growth response 1 (EGR1) is a zinc‐based transcriptional factor that is closely related to cell differentiation, proliferation, migration and invasion, and cell autophagy.^31^, ^32^ EGR1 plays an important role in smoke‐induced chrotructive through promoting cell apoptosis and autophagy.^33^ High EGR1 expression occurs in HCC tissues and cells, and via binding to LC3 promoter in HCC cells, overexpressed EGR1 was able to promote the cell autophagy induced by hypoxia.^34^ Moreover, EGR1 is also reported to contribute radio‐resistance in HCC through regulating irradiation‐induced autophagy via sponging Atg4B.^35^ This study found that EGR1 expression was high in Hep3B/So cells. In both in vitro and in vivo experiment, silencing SNHG16 inhibited the expression of EGR1, and miR‐23b‐3p inhibitor promoted EGR1 expression that partially offset the effect of down‐regulating EGR1 expression caused by SNHG16 inhibition, moreover, the cell autophagy level reflected by LC3II/LC3I expression level was similar to cell apoptosis. The findings suggested the potential relationships among SNHG16, miR‐23b‐3p, and EGR1 in sorafenib‐resistant Hep3B cells.

In conclusion, this study demonstrated the role of SNHG16 in promoting a stronger sorafenib resistance in Hep3B cells through regulating autophagy in Hep3B cells. Moreover, the mechanism is realized through regulating the expression of miR‐23b‐3p via sponging EGR1.

## CONFLICT OF INTEREST

The authors declare no conflicts of interest.

## AUTHORS’ CONTRIBUTIONS

Substantial contributions to conception and design: ZJ, XY. Data acquisition, data analysis and interpretation: XM, XH, LG. Drafting the article or critically revising it for important intellectual content: WY, JS, GC. Final approval of the version to be published: All authors agreement to be accountable for all aspects of the work in ensuring that questions related to the accuracy or integrity of the work are appropriately investigated and resolved: LG.

## Data Availability

The analyzed datasets generated during the study are available from the corresponding author on reasonable request.
